# Rate of forgetting is independent of initial degree of learning

**DOI:** 10.3758/s13421-021-01271-1

**Published:** 2022-01-06

**Authors:** Karim Rivera-Lares, Robert Logie, Alan Baddeley, Sergio Della Sala

**Affiliations:** 1 Human Cognitive Neuroscience, Psychology Department, University of Edinburgh, Edinburgh, UK; 2 Department of Psychology, University of York, Heslington, York, UK

**Keywords:** Long-term Forgetting, Repeated Testing, Forgetting Rates

## Abstract

It is commonly assumed that the rate of forgetting depends on initial degree of learning. Hence, comparison of forgetting across groups is usually carried out equating initial performance. However, these matching procedures add confounding variables. In four experiments, following Slamecka and McElree (1983, Exp 3), we challenge this assumption through manipulating initial acquisition by varying the number of presentations of the material and studying the effect on rate of subsequent forgetting. A set of 36 sentences was presented either visually or auditorily. Different participants were exposed to the material two, four or six times. Forgetting was measured by means of a cued recall test at three time-intervals (30 s, 1 day and 1 week in experiments 1 and 2; 30 s, 1 day, and 3 days in experiments 3 and 4). A different subset of 12 sentences was tested at each delay. The outcome of these experiments showed that the initial acquisition depends on number of learning trials. However, the rate of forgetting proved to be independent of initial acquisition. This pattern remains constant across modalities of presentation and of the number of learning trials. The conclusion is that forgetting does not depend on initial acquisition.

**Supplementary Information:**

The online version contains supplementary material available at 10.3758/s13421-021-01271-1.

We know that if we learn more, we will remember more (Bahrick et al., 1975; Carpenter et al., 2008; Slamecka & McElree, 1983). However, it is unclear whether the forgetting slopes following initial learning vary with the amount of initial learning. Yang et al. (2016) maintained that more exposures during learning phase would slow forgetting at shorter intervals. On the other hand, Meeter et al. (2005) found that participants with greater exposure to news did show a higher degree of learning, but not a different rate of forgetting. Kauffman and Carlsen (1989) concluded that prior knowledge of music affected the participants’ learning degree, but not their rate of forgetting. McBride and Dosher (1999) reported similar forgetting rates between conscious and automatic memory estimates using Jacoby’s (1991) dissociation procedure. Moreover, some factors, such as age, seem to affect the initial degree of learning, but not the forgetting curves for name-face pairs (e.g., Hulicka & Weiss, 1965), words (e.g., Bäckman & Mäntylä, 1988), stories (e.g., Hultsch et al., 1984), and line drawings (e.g., Rybarczyk et al., 1987).

A simple way of manipulating initial degree of learning is to vary the number of exposures to the material between participants during encoding. Slamecka and McElree (1983) used this procedure in three experiments with lists of words, associated word pairs, and sentences. They varied the number of study trials, and tested participants at three time-intervals: after a 30 s distraction task, after 1 day, and after 5 days from the study phase. All participants were tested individually and in person. Performance was measured using free recall, associative matching, cued recall, and semantic recognition. They found that a higher number of repetitions of the material increased the initial learning but did not affect the forgetting slopes.

Slamecka and McElree’s (1983) study was followed by a series of commentaries which challenged their conclusion. Loftus (1985) suggested that a monotonic scaling of the dependent variable (e.g., squared root of correct responses) in Slamecka and McElree’s study would lead to the conclusion that higher degree of learning is associated with slower forgetting. His method consisted of comparing the amount of information lost after the compared groups had reached the same number of correct items. Slamecka and McElree operationalised forgetting as the number of items forgotten between subsequent intervals, whilst Loftus defined forgetting as the amount of time required for memory performance to fall from any given level to some other level. Slamecka (1985) argued that Loftus’s method did not consider the confound of the age of the memories, that is the variation in time between initial encoding and subsequent retrieval. Suppose that the group with high degree of learning remembered 10 items at the first recall, and the group with low degree of learning remembered 8. In the Loftus method, one would have to compare the number of items lost, starting from when both groups remember 8 items. This would imply a comparison between memories of different ages for both groups. Further support for the vertical comparison used by Slamecka and McElree can be found in Wixted (2004, 2010). Wixted mentioned that the forgetting curves are characterised by an ever decreasing proportional rate of decay, which is a property of forgetting that should be expected if memories consolidated with time. This property is also implied by Jost’s second law of forgetting (1897) and by Ribot's law of retrograde amnesia (1881). Jost’s second law of forgetting states that two memories of the same strength but different ages, the older will decay more slowly than the younger. Ribot’s law of retrograde amnesia states that the temporal gradient of retrograde amnesia implies that, as they age, memories become more resistant to the effects of brain damage. To avoid comparing memories of different ages and of possible different strengths, the present study will use the definition of forgetting given by Slamecka and McElree, and therefore, their vertical comparison of forgetting rates.

Many studies on group differences in forgetting assume that there is an interaction between forgetting rates and initial level of acquisition. In these studies, the forgetting rates are compared after equating initial degree of learning for different groups (e.g., Butler et al., 2007; Muhlert et al., 2010). The same level of initial acquisition has been achieved by exposing the groups to a different number of study trials, different number of items, or using intervals of different durations (Shuell & Keppel, 1970). However, matching at learning phase does not guarantee equal learning efficiency (Zerr et al., 2018). The consequences of these manipulations on forgetting are still under debate (Elliott et al., 2014). If the initial degree of learning does not influence the forgetting rates, studies on group differences in forgetting would not need to equate initial acquisition, or they could interpret their results with the assumption that different numbers of initial exposures to the material will not impact the forgetting rates across different groups.

Testing long-term forgetting rates requires repeated testing of the same participant on multiple occasions (Baddeley et al., 2019). Using the same material at each assessment could result in a testing effect (Pan & Rickard, 2017; Roediger III & Butler, 2011), which refers to the enhancement of memory performance due to repeated tests. One way to avoid the testing effect, would be to assess a different subset of the studied material at each retention interval (Baddeley et al., 2021). Slamecka and McElree (1983) used this procedure in Experiment 3, by testing each participant with a different third of the original material at each time-interval.

Since Slamecka and McElree’s (1983) paper, to our knowledge, no attempts have been made to replicate their work despite the importance of their method for cross-group comparisons, such as those carried out in studies investigating the group differences in forgetting rate (e.g., Isaac & Mayes, 1999). Some studies have investigated forgetting rates by splitting participants into groups depending on their previous knowledge (e.g., Meeter et al., 2005). This implies that the newly acquired information relies heavily on information already stored in long term memory. It is important to investigate rates of forgetting by measuring memories of the same age, after the acquisition of new material not based on previous knowledge.

Slamecka and McElree (1983) argued that theories of forgetting neglected the problem of normal forgetting, since no theory or model could predict the effect of degree of learning on the forgetting rates. In 1963, Underwood and Keppel stated that the interference theory predicted that the higher the degree of learning, the slower should be the forgetting rate. Although Slamecka and McElree’s findings contradicted this assumption, the authors did not consider it appropriate to try explaining their results in the light of interference theory, which was undergoing significant changes. In a later review, Wixted (2010) proposed that ordinary mental exertion and memory formation interfere with the process of consolidation of new memory traces. If memories consolidate, we should expect to see a negatively accelerated function of forgetting, as it has been consistently found since Ebbinghaus (1885/1964). However, no extant theory of forgetting can explain parallel forgetting slopes that are negatively accelerated starting from different initial degrees of learning. Before beginning to theorise, we need to gather empirical data to establish what the phenomena are. This study is an attempt to look at fresh data, to test if the forgetting pattern found by Slamecka and McElree’s is robust enough to be replicated and extended.

To further explore whether initial degree of learning determines the rate of forgetting, in the current series of experiments we extended the work of Slamecka and McElree by using a larger range of number of repetitions, varying the length of the retention intervals, and using two different modalities of presentation. We also increased the generalisability and robustness of the findings by testing participants speaking two different languages, and by using two forms of remote testing. Advances in technology have made it possible to exert more control over the presentation of the material compared to that available when Slamecka and McElree carried out their study. They asked their participants to read the material on cards and switch manually through them. Instead, we used an automated presentation. In addition, recent advances in statistical methods have enabled us to analyse the data with more precision, by applying Generalised Linear Mixed Effects Models (GLMM). This holds an advantage over ANOVAs, as it does not require the averaging of data, and allows for the multilevel structure of the data.

## The Present Study

The present study is based on Experiment 3 by Slamecka and McElree (1983), with some modifications to extend the earlier study. Since we did not have access to their material, we created an original set of subject-verb-object sentences in the same fashion. After a pilot study, we decided to use 36 sentences instead of 48 as in Slamecka and McElree’s study, as performance was already very low at the first testing interval even with such reduced load. Slamecka and McElree used three and four repetitions of the material. However, we decided to explore a broader range of levels of initial degree of learning by using two, four, or six repetitions of the set of sentences. In four experiments, we varied the lengths of the intervals and the modality of presentation.

Testing the same individual with the same material on multiple occasions is likely to reduce forgetting due to the repeated testing effect (Pan & Rickard, 2017). To measure forgetting without the influence of the testing effect, we tested each participant on the material using a different subset of 12 sentences at each interval. Sampled testing, however, can also affect performance at subsequent tests either causing retrieval-induced forgetting (Anderson et al., 1994) or retrieval-induced facilitation (Baddeley et al., 2019). Retrieval-induced forgetting emerges when memories associated to a common cue compete during retrieval (Anderson et al., 1994) and retrieval-induced facilitation can occur when the associations between items is strong, such as in prose material (Baddeley et al., 2019). The material in the present study has a very low degree of integration because each sentence is independent of the rest, so the associations between the materials tested over subsequent delays are minimised. Using this kind material, neither facilitation nor inhibition has previously been found (Baddeley et al., 2021). All participants provided written, informed consent before participation and were reimbursed for their time upon completion. All were native speakers of the tested language (English or Spanish), with normal or corrected-to-normal vision, and normal hearing. This study was approved by the School of Philosophy Psychology and Language Sciences Research Ethics Committee, at the University of Edinburgh. In Experiment 3, Slamecka and McElree (1983) chose a sample of 18 participants per group. Based on this, we decided to round the sample up and use 20 subjects per group. Participants were paid for their participation between £4.00 and £6.00.

All participants were tested individually. The first recall was tested in person in all experiments. In Experiments 1 and 2 the second and third recall was tested by email, and in Experiments 3 and 4 recall was tested by telephone. For all experiments, we used cued recall, with the subject of the sentence as cue.

## Planned analyses

Slamecka and McElree (1983) conducted their analyses using ANOVA. However, Jaeger (2008) suggested that non-normal data, such as those obtained both in this study and in Slamecka and McElree’s study, violate the assumptions of normality needed to perform ANOVA. Logistic regression is recommended to handle binomial outcomes assuming that the observations are independent (Bye & Riley, 1989). In the present study, all participants were tested at the three retention intervals violating the assumption of non-independence. These nested data are non-independent such that the responses on the dependent variable from participants in the same group are more similar than would be expected by chance (Bliese, 2000). Mixed Effects models can account for this non-independence and thus account for the multi-level structure of the data in such a way that it would not need to be averaged as in ANOVA, avoiding the loss of information (Bliese et al., 2018). Instead, Mixed-effects models include random effects of participants and items, meaning that the model takes into account the variance in the data explained by the different memory capacity of the participants and the different difficulty of the items. This consequently allows for clearer insights into forgetting over time than traditional analyses such as ANOVAs as these tend to be confounded by individual differences in participant's memory capacity or differences in item difficulties.

For the analyses, the dependent variable was the binary outcome correct (1) or incorrect (0) response per sentence per participant. Correct responses were defined as recalling the verb or the noun that corresponded to the subject presented as cue in the cued recall task. We used a Bernoulli data distribution. To account for the multi-level structure in the data (i.e., individuals were measured repeatedly; items were used in all number of repetitions and at each retention interval), we modelled a random intercept (over both items and subjects), and random effect of the retention interval (over both items and subjects), and a random effect of the number of repetitions over items. The number of repetitions was a between-subjects factor, so it was included only as a fixed effect over subjects in the model. The data were analysed using Bayesian generalised linear mixed models, fitted employing the Stan modelling language (Carpenter et al., 2017) and the R package *brms* (Bürkner, 2017, 2018) using the default priors. Parameter uncertainty is described by the 95% credible interval (CI) of the posterior distribution in addition to the mean parameter value. Substantial in the context of Bayesian inference, means that 0 is not within the boundaries of the 95% CI.

## Experiment 1

### Method

#### Participants

Sixty students from the University of Edinburgh (*M*_*age*_ = 22.25, *SD* = 2.77, range: 19-30, 13 men) participated in this experiment. Two participants did not respond to the invitation to complete the second and third tests, so their data were discarded. Both participants were substituted.

#### Materials

The materials employed in this experiment were 36 subject-verb-object sentences written in English. Each subject, verb and object was used only once. The sentences were constructed using verbs that were not commonly associated with the subject to minimise guessing. For example, we did not use “the teacher taught the lesson”, rather we used “the teacher ate the bread”. The complete set of sentences is given in the Supplementary Material, Appendix A.

Memory was tested with cued recall in written form (see Baddeley et al., 2019), using three different response sheets. Considerable evidence has been accrued showing that repeated retrieval of encoded material enhances learning (review in Roediger III & Butler, 2011). To minimise these practice effects at each testing phase, only a subset of the sentences was tested at any given delay (Baddeley et al., 2019; Stamate et al., 2020). Hence, each response sheet contained one of three subsets of 12 sentences taken from the 36 sentences presented in the study trials. The order of the items in each response sheet was fixed. In each response sheet, there was a list of the subjects of the sentences, each one followed by a line in which the participants were asked to write down the verb and direct object that correctly completed the sentence.

To minimise the effects of repeated retrieval in each successive test, the 36 sentences were independent from one another (i.e., they do not form part of a narrative nor can be arranged in any coherent manner). The subjects of each sentence were used as cues for the cued recall test.

#### Procedure

The participants sat in front of the computer at a comfortable distance from the screen. They were told that they were about to read some sentences on the screen, and that they should memorise the sentences for further test. They were informed that the sentences were going to be presented more than once, so that they would have more than one opportunity to learn them, but they were not informed on how many times they would see each sentence. Every participant practiced the distractor task once, starting from 100. After this practice, the researcher clarified any questions from the participants and then the first study trial commenced. Each study trial consisted of the presentation of the 36 sentences on a computer screen, written with black letters on a white background. Between each study trial, the screen remained blank for 15 s. Within each study trial, the sentences were presented one by one. Each sentence was presented for 5 s, followed by a 2 s interval during which the screen was blank. The sentences were presented in a different order at each study trial. Two seconds after the last sentence of the last study trial was shown, the instructions for a distractor task were presented on screen, asking participants to perform subtractions by sevens from a three-digit number. After 30 s, the screen showed the word “stop”, indicating the end of the presentation.

Following the presentation of the last study trial and the completion of the distractor task, each participant was presented with the first response sheet. They were asked to try to retrieve the sentences for at least 5 minutes, with no upper limit, and to leave the response field blank if they could not remember the correct answer. Before they left, they were reminded that they would receive an email with the following response sheet the next day and a week later. We tested performance at the second and third time-intervals via email to remove the possibility that participants would fail to attend the three sessions. However, we tested nine participants in person as a control to ensure that testing via email did not decrease performance when compared to in-person testing.

The 60 participants were divided into three groups of 20 participants each, and each one of these groups was presented with two, four or six study trials. All participants were tested at three intervals – 30 s, 24 hr, and 1 week after the last study trial – using a different response sheet at each interval. These response sheets were counterbalanced across all conditions.

### Results

**Fig. 1:**
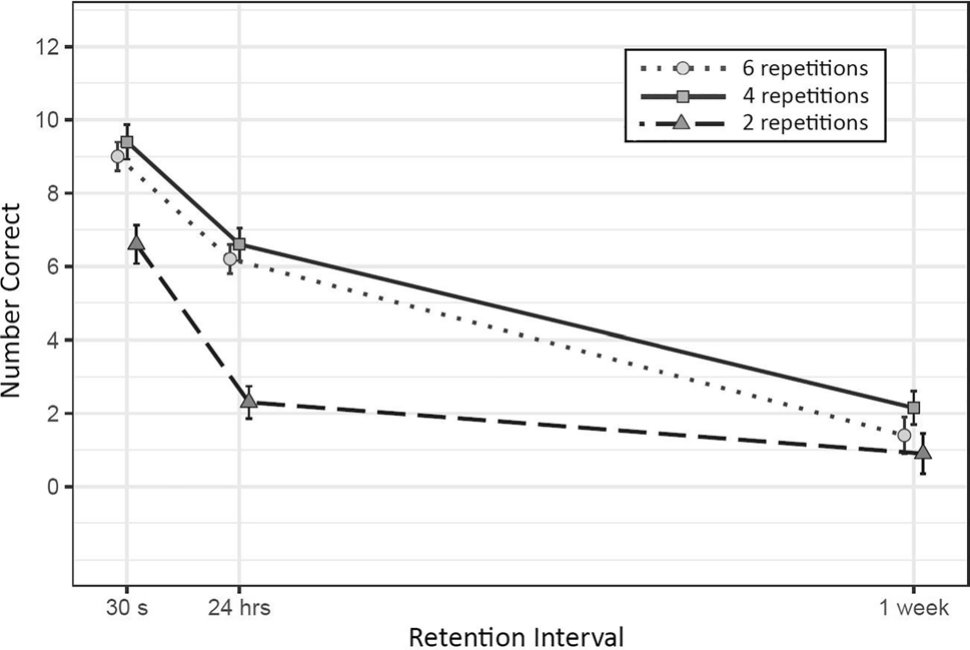
Mean and Standard Errors of Correct Responses at Each Combination of Number of Repetitions and Retention Interval

The forgetting rate of participants tested always in person was not substantially different from that of the participants tested via email. Means and standard errors of number of correct items recalled at each retention interval are reported in Fig. 1.

#### Effect of retention interval

There was clear evidence of the retention interval effect between 30 s and 24 hr (*b* = -1.68, *SD* = 0.34, CI = [-2.37, -1.03]), between 30 s and 1 week (*b* = -4.33, *SD* = 0.46, CI = [-5.27, -3.46]), and between 24 hr and 1 week (*b* = -2.65, *SD* = 0.43, CI = [-3.53, -1.84]).

#### Effect of number of repetitions

As can be seen from Fig. 1, the difference between six and four repetitions was not substantial (*b* = 0.28, *SD* = 0.76, CI = [-1.2, 1.77]). We found substantial evidence for an effect of number of repetitions between four and two (*b* = -1.73, *SD* = 0.73, CI = [-3.22, -0.36]) and between six and two (*b* = -1.45, *SD* = 0.72, CI = [-2.83, -0.14]).

#### Interaction effects

As can be seen from Fig. 1, the probability of correctly retrieving an item depends mostly on main effects, rather than on the combinations of number of repetitions and retention intervals. The combination of number of repetitions and delay resulted in nine interactions to compare. Only three of these interactions showed to be substantially different: the difference between six and two repetitions from 30 s to 24 hr (*b* = -1.02, *SD* = 0.48, CI = [-1.96, -0.1]), the difference between four and two repetitions from 24 hr to 1 week (*b* = 1.36, *SD* = 0.6, CI = [0.23, 2.55]), and the difference between six and two repetitions from 24 hr to 1 week (*b* = 1.66, *SD* = 0.6, CI = [0.5, 2.85])^1^. Floor effects after the 1 week interval might have concealed possible evidence of faster forgetting in longer delays with a lower number of initial repetitions. Mean performance was at floor at the 1 week delay for the group with two repetitions, with 9 out of 20 participants scoring zero.

The rest of the possible interactions were not substantially different, namely the slopes from 30 s to 24 hr with six and four repetitions (*b* = -0.18, *SD* = 0.45, CI = [-1.11, 0.71]) and four and two repetitions (*b* = -0.84, *SD* = 0.5, CI = [-1.8, 0.13]); the slopes from 24 hr to 1 week with six and four repetitions (*b* = 0.31, *SD* = 0.56, CI = [-0.79, 1.42]), and the slopes between 30 s and 1 week with six and four repetitions (*b* = 0.13, *SD* = 0.61, CI = [-1.05, 1.33]), with four and two repetitions (*b* = 0.51, *SD* = 0.63, CI = [-0.68, 1.8]), and with six and two repetitions (*b* = 0.64, *SD* = 0.62, CI = [-0.56, 1.85]).

#### Errors

The most common errors were omissions of verbs and nouns from the presented sentences; these errors increased in frequency after delays, and were very similar between four and six repetitions, but increased with two repetitions. Some intrusions were also recorded; they also slightly increased with time (see Supplementary Material, Appendix B, Figures S1 and S2). At one week, there were slightly more omissions for six and two repetitions than for four repetitions.

#### Stringent scoring

A more stringent scoring was also carried out by considering correct only those responses in which both verb and noun were correct. As in the more lenient scoring, we found substantial evidence of forgetting at all time-intervals; we found no difference between six and four repetitions at 30 s, but the difference between two and four repetitions, and two and six repetitions was substantial. Three out of nine comparisons between forgetting slopes were substantial: the one between six and four, and six and two repetitions between 1 day and one week, and the one between six and two repetitions between 30 s and one week. The interactions that were substantially different were the same for the lenient scoring, except for the difference between six and four repetitions between 30 s and 24 hr. This interaction was no longer substantial, but a new one emerged between the same repetition groups, this time between 30 s and one week. In other words, with the stringent scoring, all the substantially different slopes included the group with two repetitions at 1 week. The results of the analysis are now reported in the Supplementary Material, Appendix C.

Partially correct responses (i.e., when only the verb or only the object were correct) were calculated as a percentage of the total number of responses at each group of number of repetitions. The frequency of these partially correct responses was low, with 5% as the highest percentage in this experiment.

### Discussion

There was a substantial difference between the initial degree of learning across conditions with two repetitions and those with four or six repetitions. No difference in initial acquisition was found between four and six repetitions. We observed faster forgetting at shorter delays, and slower forgetting at longer delays. The lack of interaction between the initial degree of learning and the retention intervals indicates that the rate of forgetting does not depend on initial level of acquisition.

This experiment differs from the original work of Slamecka and McElree (1983) by exploring a broader number of repetitions. We used six, four, and two repetitions, compared to their study which used three and four repetitions. However, we found no difference in performance at 30 s between four and six repetitions.

Out of nine possible comparisons between two slopes, only three were reliably different. These slopes were the ones between 24 hr and 1 week when comparing four and two, and six and two repetitions, and the ones between 30 s and 24 hr with six versus two repetitions. As can be seen in Fig. 1, the data from the 1 week retention interval was at floor, which complicates the interpretation of these results. Two of the three relevant interactions resulted from comparisons that included the data with two repetitions at 1 week. These data points create a rather flat slop which is the result of very little room for forgetting, thus the supposed interaction cannot be interpreted as a slower rate of forgetting due to a difference in the initial degree of acquisition.

These results are comparable with those obtained by Slamecka and McElree (1983) in that despite having two degrees of initial learning, the forgetting slopes did not vary. Participants with two repetitions correctly recalled two items on average after one day, which leaves very little space for forgetting. So, it is important to ensure that the lack of a difference in forgetting rates for different initial levels of learning is sufficiently robust to replicate. We addressed this using the same paradigm but with auditory presentation in Experiment 2. Testing forgetting curves after auditory presentation would also increase the ecological validity of our findings, since verbal information in real life is presented not only visually but also auditorily.

## Experiment 2

### Method

#### Participants

Sixty students from the University of Edinburgh (*M*_*age*_ = 22.35, *SD* = 3.5, range: 18 to 30, 14 men) participated in this experiment. None had taken part in Experiment 1.

#### Materials

The materials were the same 36 sentences in English used in Experiment 1. This time, the sentences were recorded by a professional broadcaster. The sentences were edited with Audacity (2020) version 2.3.3, a recording and editing software package. As in Experiment 1, the order of the sentences was randomised across study trials.

#### Procedure

The participants sat comfortably in a chair, with headphones on. The headphones were plugged in into the computer where the recordings were played. The participants were asked to turn off the sound and vibration of their mobile phones. The volume of the computer was adjusted until it was comfortable for the participant. The participants were informed that they would listen to the sentences more than once and that some silences would seem longer, but the learning phase was not going to be over until indicated by the experimenter who was looking at the soundwaves produced by the recordings on the screen of the computer. The participants practiced the distractor task, and then started the learning phase.

Each participant was presented with either two, four, or six study trials, followed by the instruction to perform the distractor task. The sentences were presented at a rate of approximately 2.5 s per sentence, with 2 s silence between sentences, and 15 s silence between study trials. Once the completed the distractor task, they were asked to complete the first response sheet. The response sheets used for this experiment were the same as in Experiment 1. As in the previous experiment, participants were asked to try to remember for at least five minutes if they have not completed all the sentences, and to leave the response field blank if they could not remember the answer. Performance was tested at 30 s, 24 hr, and 1 week. The order of the items on the response sheets was fixed for each participant.

At the end of the session, the participants were reminded that they would receive an email with the following response sheet the next day and a week later. As in Experiment 1, we tested performance in the longer intervals via email to remove the possibility that participants would fail to attend the remaining sessions, except for nine participants who were tested in person as a control to ensure that testing via email would not decrease the scores compared to in person testing.

### Results

**Fig. 2:**
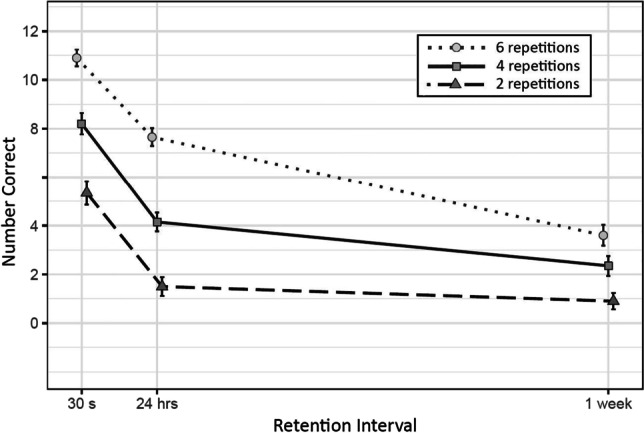
Mean and Standard Errors of Correct Responses at Each Combination of Number of Repetitions and Retention Interval

The forgetting rate of participants tested completely in person was not substantially different from the participants tested via email. Means and standard errors of number of correct items recalled at each retention interval are depicted in Fig. 2.

#### Effect of retention interval

There was substantial evidence of the effect of time between 30 s and 24 hr (*b* = -2.46, *SD* = 0.43, CI = [-3.33, -1.66]), between 30 s and 1 week (*b* = -4.45, *SD* = 0.49, CI = [-5.48, -3.53]), and between 24 hr and 1 week (*b* = -1.99, *SD* = 0.38, CI = [-2.77, -1.27]).

#### Effect of number of repetitions

There was substantial evidence of the effect of number of repetitions between six and four ((*b* = -2.00, *SD* = 0.55, CI = [-3.13, -0.95]), between six and two (*b* = -3.00, *SD* = 0.56, CI = [-4.49, -2.24]), and between four and two (*b* = -1, *SD* = 0.48, CI = [-2.27, -0.39).

#### Interaction effects

There was no substantial evidence of an interaction between the number of repetitions and retention interval^2^ in the delays from 30 s to 24 hr with six and four repetitions (*b* = 0.23, *SD* = 0.51, CI = [-0.77, 1.26]), with four and two (*b* = -0.1, *SD* = 0.46, CI = [-1, 0.79]), or with six and two (*b* = 0.13, *SD* = 0.55, CI = [-0.93, 1.23]). The same was true for the delays between 24 hr and 1 week between six and four repetitions (*b* = 0.52, *SD* = 0.56, CI = [-0.58, 1.59]), between four and two (*b* = 0.62, *SD* = 0.63, CI = [-0.65, 1.86]), or between six and two (*b* = 1.14, *SD* = 0.62, CI = [-0.09, 2.35]), and for the delays from 30 s to 1 week with six and four (*b* = 0.76, *SD* = 0.64, CI = [-0.48, 1.99]), four and two (*b* = 0.51, *SD* = 0.63, CI = [-0.74, 1.75]), or six and two (*b* = 1.27, *SD* = 0.67, CI = [-0.05, 2.61]).

#### Errors

The most common errors were omissions of verbs and nouns from the presented sentences; these errors were more frequent with less repetitions and after delays (see Supplementary Material, Appendix B, Figures S3 and S4). Some intrusions were also recorded; they also slightly increased with the exception of nouns with two repetitions, in which intrusions of studied material decreased slightly after one week.

#### Stringent scoring

As in Experiment 1, we carried out an analysis with a stringent scoring in which both verb and noun needed to be correct. We found substantial evidence of the retention interval at all time intervals, and substantial evidence of the difference between six and four, four and two, and six and four repetitions. No interactions were found to be substantially difference (see Supplementary Material, Appendix C, page 11).

Partial responses were infrequent, with the highest percentage being 3.19%.

### Discussion

We found reliable evidence of a decrement in performance with each subsequent retention interval. In Experiment 1, there was no difference in performance at 30 s between four and six repetitions. In Experiment 2 we found that the initial level of acquisition increased with the number of repetitions. Importantly, the degree of learning did not influence the rate of forgetting. As in Experiment 1, we found faster forgetting in the shorter interval compared to the longer interval.

One issue remains, and that is the possible impact of floor effects after a delay of 1 week. These might have obscured possible evidence for faster forgetting over this longer interval with fewer initial repetitions. Given that performance for two repetitions was above floor for delays of 24 hr, there is a possibility that performance will also be above floor for delays of less than 1 week, thereby allowing for possible evidence of faster forgetting over delays longer than 24 hr but shorter than 1 week. Therefore, in Experiment 3, we carried out a replication of Experiment 2 with auditory presentation, reducing the 1 week interval to 3 days. Because we found a difference in performance at immediate test amongst the three number of repetitions with auditory presentation, these conditions remained the same in Experiment 3.

## Experiment 3

### Method

#### Participants

Sixty young Mexican adults (*M*_*age*_ = 21.77, *SD* = 3.28, range: 18-30, 14 men) were recruited by word of mouth. All participants had a minimum of 13 years of education.

#### Materials

To create the materials for this experiment, we translated the 36 sentences used in the previous two experiments into Spanish, which was the native language of the participants and of the experimenter. We slightly changed some of the sentences to make them culturally appropriate. The presentation of the materials was identical to that in Experiment 2. A complete list of the materials is given in the Supplementary Material, Appendix A.

#### Procedure

The auditory presentation of the sentences was identical to that of Experiment 2. Participants were presented with two, four, or six study trials of recorded audio versions of the 36 sentences in Spanish used in Experiment 2, at a rate of 2.5 s per sentence, with 2 s silence between sentences, and 15 s silence between study trials. The order of the sentences was randomised across study trials.

For the testing phase, as in the previous experiments, three blocks of responses were created using a different subset of 12 sentences for each block. In each block, the subject of the sentence was used as a cue. The order of the items in each block was fixed. All participants were tested on the whole material, and the order in which the blocks or responses were tested at the three delays was counterbalanced across all conditions.

Performance was tested in person using a response sheet for the first time-interval. The second and third recalls were assessed by telephone for the following reasons: first, remote testing prevents participants from not completing the experiment due to inability or unwillingness to attend the testing space three times. Second, it gives us the possibility of replicating this study in the future with clinical populations, or with populations who cannot attend to the face-to-face testing more than once due to mobility problems. Third, the social distancing measures adopted during the COVID-19 pandemic rendered testing in person impossible. Testing via telephone has been shown to be an appropriate method of assessing long-term forgetting (Allen et al., 2019) and has been used in studies with similar paradigms to the one in the present study (e.g., Stamate et al., 2020).

For the telephone testing, the experimenter called the participants at an agreed time. The experimenter read the subjects of the phrases one by one, giving time to the participants to answer. Participants were asked to try to retrieve the sentences for at least 5 minutes, with no upper limit, and to verbally indicate if they could not remember a phrase, instead of guessing. After a first round of reading the subjects of the phrases one by one, the experimenter repeated to the participant the subjects of the phrases to which the participant had not responded.

Performance was assessed at intervals of 30 s, 24 hr, and 3 days to investigate possible differences in forgetting rates in intervals longer than 24 hr, but shorter than 1 week. To further ensure that the remote testing did not differ from the in-person testing, a group of 12 participants were tested in person across all intervals. This group received only four repetitions of the sentences.

All participants were tested at intervals of 30 s, 24 hr, and 3 days. We changed the 1 week interval of Experiment 2 to 3 days to avoid the floor effects found in said experiment.

### Results

**Fig. 3:**
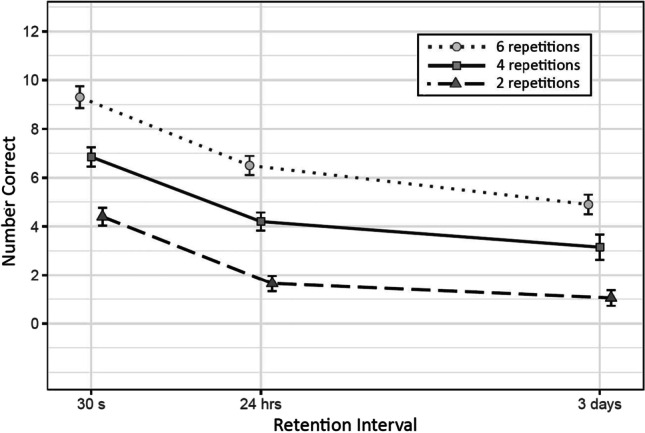
Mean and Standard Errors of Correct Responses at Each Combination of Number of Repetitions and Retention Interval

Means and standard errors of number of correct items recalled at each retention interval can be seen in Fig. 3.

#### Effect of retention interval

We found substantial evidence of a retention interval effect between 30 s and 24 hr (*b* = -1.2, *SD* = 0.28, CI = [-1.73, -0.64]), between 30 s and 3 days (*b* = -2.04, *SD* = 0.35, CI = [-2.75, -1.38]), and between 24 hr and 3 days (*b* = -0.85, *SD* = 0.33, CI = [-1.55, -0.2]).

#### Effect of number of repetitions

There was substantial evidence of an effect of the number of repetitions between six and four repetitions (*b* = -1, *SD* = 0.34, CI = [-1.81, -0.48]), between six and two repetitions (*b* = -2, *SD* = 0.34, CI = [-2.83, -1.48]), and between four and two repetitions (*b* = -1, *SD* = 0.34, CI = [-1.68, -0.36]).

#### Interaction effects

There was no substantial evidence of the interaction between number of repetitions and retention interval from 30 s to 24 hr between six and four repetitions (*b* = 0.06, *SD* = 0.36, CI = [-0.66, 0.77]), between six and two repetitions (*b* = -0.5, *SD* = 0.39, CI = [-1.31, 0.24]), or between six and two repetitions (*b* = -0.45, *SD* = 0.42, CI = [-1.31, 0.33]). We did not find substantial evidence either in the intervals from 24 hr to 1 week between six and four repetitions (*b* = 0.2, *SD* = 0.46, CI = [-0.71, 1.12]), between four and two repetitions (*b* = -0.08, *SD* = 0.53, CI = [-1.13, 0.95]), or between six and two repetitions (*b* = 0.12, *SD* = 0.53, CI = [-0.98, 1.16]); nor over the retention interval from 30 s to 1 week with six and four repetitions (*b* = 0.26, *SD* = 0.47, CI = [-0.65, 1.17]), between four and two repetitions (*b* = -0.59, *SD* = 0.51, CI = [-1.6, 0.4]), or between six and two repetitions (*b* = -0.33, *SD* = 0.5, CI = [-1.34, 0.65]).

#### Errors

The most frequent errors were omissions of verbs and nouns from the presented sentences; these errors were more frequent with less repetitions and after delays, although the difference was minimal between one day and three days with two repetitions. There were some intrusions, which increased with each delay with six repetitions but remained the same or slightly decreased with four and two repetitions (see Supplementary Material, Appendix B, Figures S5 and S6).

#### Stringent scoring

When the data was scored in a more stringent manner, the same patters were found: substantial evidence of time interval at all intervals, effect of number of repetitions, and no substantial evidence of interactions (see Supplementary Material, Appendix C, page 12). Partial responses were infrequent with a maximum of 3.19%.

### Discussion

Performance was higher at 30 s with more repetitions and decreased at each subsequent retention interval. No interaction between the number of repetitions and time-interval was found. Faster forgetting occurred in the short delay compared to the long delay.

As in Experiment 2, Experiment 3 used auditory presentation of the sentences. The results from both experiments were similar except that for Experiment 3 the participants performed slightly worse at 30 s. In Experiment 2, participants performed at floor at the 1 week interval. In Experiment 3 the reduced interval to 3 days resulted in better performance with four and six repetitions, and a small improvement with two repetitions.

In sum, our findings have now been shown to be robust across three experiments in two different languages, and with both visual and auditory presentation of the to-be-remembered material. In Experiments 1 and 2, we found parallel forgetting lines regardless of the degree of initial learning. However, floor effects at the 1 week delay complicated the interpretation of the lack of interaction between time-interval and initial degree of learning. In Experiment 3, performance was off floor at the last delay using auditory presentation and showed clearly parallel forgetting rates for different degrees of initial learning. In Experiment 4, we used the same delay as in Experiment 3 but this time with visual presentation and dropping the six repetitions condition, since Experiment 1 showed no difference in initial degree of learning between four and six repetitions.

## Experiment 4

### Method

#### Participants

Forty young Mexican adults (*M*_*age*_ = 23.85, *SD* = 3.25, range: 18 to 30, 18 men) were recruited by word of mouth. All of them had a minimum of 13 years of education. None had taken part in Experiment 3.

#### Materials

We used the same 36 sentences as in Experiment 3.

#### Procedure

The encoding phase was the same as in Experiment 1, except that the six repetitions condition was dropped. The first recall was carried out as in Experiment 1. The second and third recalls were assessed by telephone following the same procedure as in Experiment 3. As in experiment 3, we tested a group of 12 participants to ensure that remote testing did not differ from the in-person testing.

### Results

**Fig. 4:**
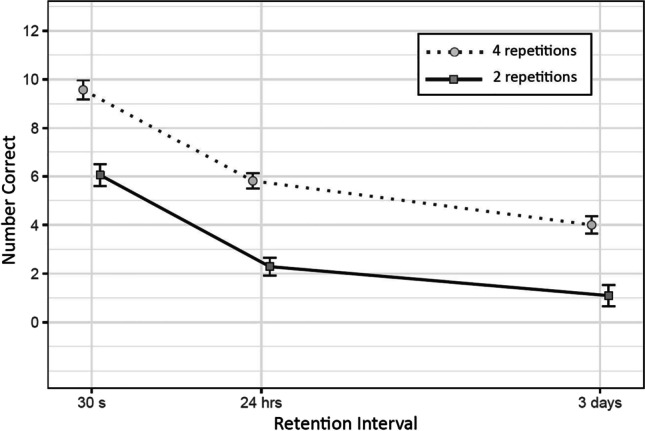
Mean and Standard Errors of Correct Responses at Each Combination of Number of Repetitions and Retention Interval

The forgetting rate of participants tested completely in person was not substantially different from the participants tested via telephone. Means and standard errors of number of correct items recalled at each retention interval can be seen in Fig. 4.

#### Effect of retention interval

There was substantial evidence of the effect of time between 30 s and 24 hr (*b* = -2.25, *SD* = 0.39, CI = [-3.03, -1.51]), between 30 s and 3 days (*b* = -3.27, *SD* = 0.41, CI = [-4.11, -2.51]), and between 24 hr and 3 days (*b* = -1.03, *SD* = 0.34, CI = [-1.69, -0.38]).

#### Effect of number of repetitions

There was substantial evidence of the effect of number of repetitions (*b* = -2, *SD* = 0.55, CI = [-3.3, -1.12]).

#### Interaction effects

There was no substantial evidence of an interaction between the number of repetitions and the time-interval from 30 s to 24 hr (*b* = -0.15, *SD* = 0.51, CI = [-1.21, 0.81]), from 30 s to 3 days (*b* = -0.1, *SD* = 0.5, CI = [-0.95, 0.99]), or from 24 hr and 3 days (*b* = 0.17, *SD* = 0.53, CI = [-0.84, 1.23]).

#### Errors

As in the previous three experiments, most errors were omissions of verbs and nouns, which were more frequent with less repetitions and more time. As before, some intrusions were present. The intrusions of not-studied material remained largely the same for verbs and nouns, whilst the intrusions of studied material increased after one day and decreased after three days for the group with four repetitions, and slightly decreased for the group with two repetitions (see Supplementary Material, Appendix B, Figures S7 and S8).

### Stringent scoring

In a stringent analysis were responses were scored correct only when the verb and the noun are correct, the same pattern was found: there was substantial evidence of an effect of time at all times, an effect of number of repetitions, and no effect of interactions (see Supplementary Material, Appendix C, page 13). Partial responses were infrequent with a maximum of 3.47%).

### Discussion

As in Experiment 1, four repetitions of the stimulus material resulted in better memory performance than two repetition at delays of 30 s, 24 hours and 3 days, and performance decreased at each retention interval. There was no difference in the rate of forgetting of both groups. As in the previous three experiments, forgetting was faster between 30 s and 24 hours than between 24 hours and 3 days.

Initial performance was comparable to that of Experiment 1 with two and four repetitions. The reduction of the length of the retention intervals kept performance of the four repetitions group off floor but was no different from the scores obtained by the group with two repetitions in Experiment 1 who were tested a week after learning.

Taken together, the results of the four experiments show that forgetting is independent for the initial degree of learning.

## General Discussion

In a series of four experiments, we investigated the relationship between initial degree of learning and the rate of forgetting of a list of sentences. Initial degree of learning was manipulated by exposing participants to two, four, or six repetitions, except for Experiment 4 in which the six repetitions condition was dropped. Memory performance was tested using cued recall at three time-intervals. In experiments 1 and 2 the intervals were 30 s, 24 hr, and 1 week. In experiments 3 and 4, the intervals were 30 s, 24 hr, and 3 days.

The manipulation of the degree of learning was successful in all experiments except for Experiment 1, in which the groups with four and six repetitions showed identical performance at 30 s. Our results shown a typical finding of long-term forgetting studies, which is also consistent with Ebbinghaus’s (1885/Ebbinghaus, 1964) forgetting curve: most of the forgetting occurred in the short intervals between 30 s and 24 hr, with slower forgetting in the intervals from 24 hr onwards.

More importantly, in all four experiments, the rate of forgetting was not modulated by the initial degree of learning, clearly replicating the findings of Experiment 3 of Slamecka and McElree (1983). Whenever we found evidence for an interaction between time and initial degree of learning, performance was too low to measure any forgetting, and so we cannot assume that such interaction reflects a real difference in forgetting related to the number of study trials. Lower performance in the two repetitions group in all experiments might be a cause for concern as there is not enough room for further forgetting. However, the consistency of the outcome in four experiments is reassuring. Especially since in Experiment 2 and 3 the forgetting rates are the same between the groups with four and six repetitions. These results contradict Loftus (1985), who suggested that the rate of forgetting is faster for groups who were exposed to fewer repetitions of the material during encoding. This difference may stem from a different operationalisation of forgetting and hence methodological approach.

Our results present a theoretical problem. No current theory of forgetting can account for forgetting that starts from different levels yet results in parallel forgetting slopes that are negatively accelerated. One possible explanation could be that there are two underlying processes shaping forgetting, one possibly based on interference and one based on the decay of the memory trace. The classic forgetting function from Ebbinghaus (1885) onwards implies a negatively accelerated function over time with different initial levels resulting in non-parallel curves. Similarly, fitting a single function to forgetting data such as Loftus (1985) proposed, also implicitly assumes a unitary source of initial trace strength. This is inconsistent with Slamecka and McElree’s (1983) and our current data, which instead suggest the need to assume two or more contributions to the initial starting point and the subsequent course of the forgetting curves. Within this frame, one source of forgetting would represent a gradual erosion of traces over time and the other is based on the assumption that different material or different types of memory traces differ in their resistance to such erosion.

The independence of the forgetting rates from the initial degree of learning has important implications to studies which use cross-group comparisons (e.g., ageing studies). These studies encounter the methodological issue of equating initial degree of learning at the expense of adding confounding variables such as measuring memories of a different age. If learning does not influence forgetting, it might be possible to carry out these experiments without the need to match the initial degree of learning, or on the other hand, that initial degree of learning can be safely matched by increasing the number of repetitions.

In our study we reported low performance at longer intervals. It has been proposed that a second exposure after initial retrieval from long-term memory improves memory performance at subsequent tests when compared to no re-exposure (Carpenter, 2012; Delaney et al., 2010; Roediger III & Butler, 2011; Roediger III & Karpicke, 2006). Research suggests that the influence that repeated testing has on recall, might depend on the integration of the material (Anderson et al., 2000; Baddeley et al., 2019; Bäuml & Hartinger, 2002; Chan, 2009; Chan, 2010). Integrated material refers to structures composed of highly interconnected items (e.g., narratives), while non-integrated material refers to independent items (e.g., words). Integrated material occurs when individual items are associated with each other as in the gist of a prose passage unlike the material used in the current study, whereby sentences are independent from one another (see also Baddeley et al., 2021). Moreover, the sentences we used in this study were constructed in a way that participants could not infer the verb and noun by reading the subject, used as a cue. As such, the material could be considered as non-integrated. Using non-integrated materials, testing can impair delayed recall of the non-tested items (Anderson, 2003; Storm & Levy, 2012; Tandoh & Naka, 2007) which could explain the lower performance at the longer intervals.

## Supplementary Information

ESM 1 (DOCX 661 kb)

## Data Availability

The datasets generated and analysed during the current study are available in the OSF repository at https://osf.io/a3t4q.

## Footnotes

1 We carried out a second analysis excluding the participants with 0 correct at 1 week. No relevant interactions were found.

2 We carried out a second analysis after excluding participants performing at floor at 1 week and again, no interactions were found to be substantial.

## Code availability

R code available in the OSF repository at https://osf.io/a3t4q.
