# Supplementary material for: Rate of forgetting is independent of initial degree of learning
Source: Mem Cognit. 2022 Jan 6;50(8):1706–18. doi: 10.3758/s13421-021-01271-1 (PMC8735725; doi:10.3758/s13421-021-01271-1)
Supplement: Supplementary file 1 — (DOCX 661 kb) [file 13421_2021_1271_MOESM1_ESM.docx]

**Supplementary Material**

**Appendix A**

Sentences used in Experiments 1 and 2.

1. The teacher ate the bread.
2. The cook bent the band.
3. The carpenter found the coin.
4. The clown learnt the recipe.
5. The firefighter won the be.
6. The sniper shook the jar.
7. The comedian made the bed.
8. The writer hit the ball.
9. The journalist froze the cake.
10. The runner stole the ring.
11. The president shot the arrow.
12. The violinist left the purse.
13. The spy squeezed the bottle.
14. The baker enjoyed the sunset.
15. The breeder split the bill.
16. The chemist took the bracelet.
17. The superhero called the neighbour.
18. The gardener threw the box.
19. The lawyer wore the gloves.
20. The client rode the bike.
21. The physicist sang the lullaby.
22. The driver closed the curtains.
23. The princess finished the desk.
24. The singer locked the chest.
25. The pilot pulled the tablecloth.
26. The actor checked the tubes.
27. The patient sold the bag.
28. The surgeon spilled the oil.
29. The hunter borrowed the plate.
30. The engineer lit the candle.
31. The butcher kicked the branch.
32. The bartender printed the label.
33. The student used the spoon.
34. The librarian wrote the invoice.
35. The assistant pushed the lamp.
36. The plumber dug the garden.

Sentences used in Experiments 3 and 4.

1. El abogado usó los guantes.
2. El actor revisó la tubería.
3. El asistente empujó la lámpara.
4. El bombero ganó la apuesta.
5. El cantante cerró el cofre.
6. El carnicero rompió la rama.
7. El carpintero encontró la moneda.
8. El cartero lavó la ropa.
9. El cirujano derramó el aceite.
10. El cliente manejó el camión.
11. El comediante tendió la cama.
12. El conductor abrió la puerta.
13. El corredor robó el anillo.
14. El doctor limpió el sótano.
15. El escritor pateó el balón.
16. El espía exprimió la naranja.
17. El estudiante colgó la pintura.
18. El granjero quemó las revistas.
19. El mago gastó el dinero.
20. El músico sacó la basura.
21. El paciente vendió la caja.
22. El panadero disfrutó el atardecer.
23. El payaso preparó el arroz.
24. El periodista congeló el pastel.
25. El piloto puso el mantel.
26. El plomero plantó el árbol.
27. El político hirvió el café.
28. El presidente disparó la flecha.
29. La princesa armó el escritorio.
30. El químico tomó la pulsera.
31. El recepcionista barrió la cocina.
32. El sastre comenzó la campaña.
33. El superhéroe tocó el timbre.
34. El terapeuta contestó el teléfono.
35. El veterinario pagó la cuenta.
36. El violinista dejó la bolsa.

**Appendix B**

Figures S1 to S8 detail the errors committed by the participants in each of the four experiments of the study. The figures show the number of correct and incorrect responses for either verbs or nouns from each experiment. The bars represent correct responses, and three types of errors. Not-studied responses refer to intrusions of verbs or nouns that were not presented during the study phase. Intrusions refers to verbs or nouns that were presented during the study phase but that were incorrectly attributed to the wrong sentence. Omitted refers to lack of response.

**Figure S1**

*Total of correctly and incorrectly recalled verbs in Experiment 1.*


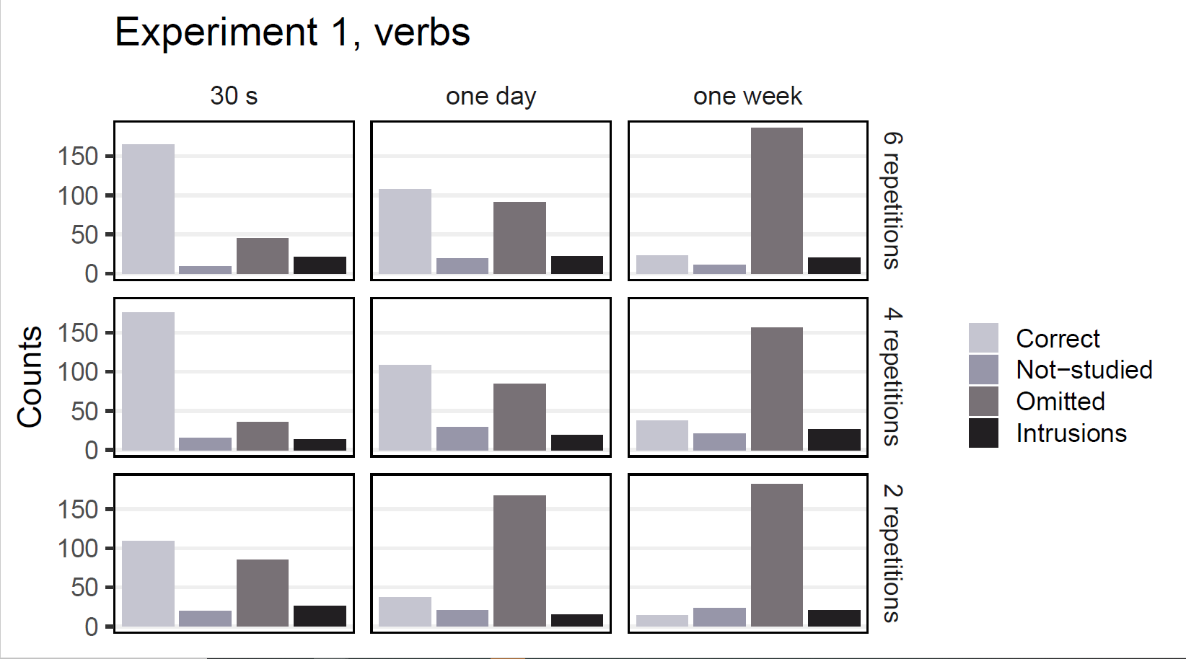


**Figure S2**

*Total of correctly and incorrectly recalled nouns in Experiment 1.*


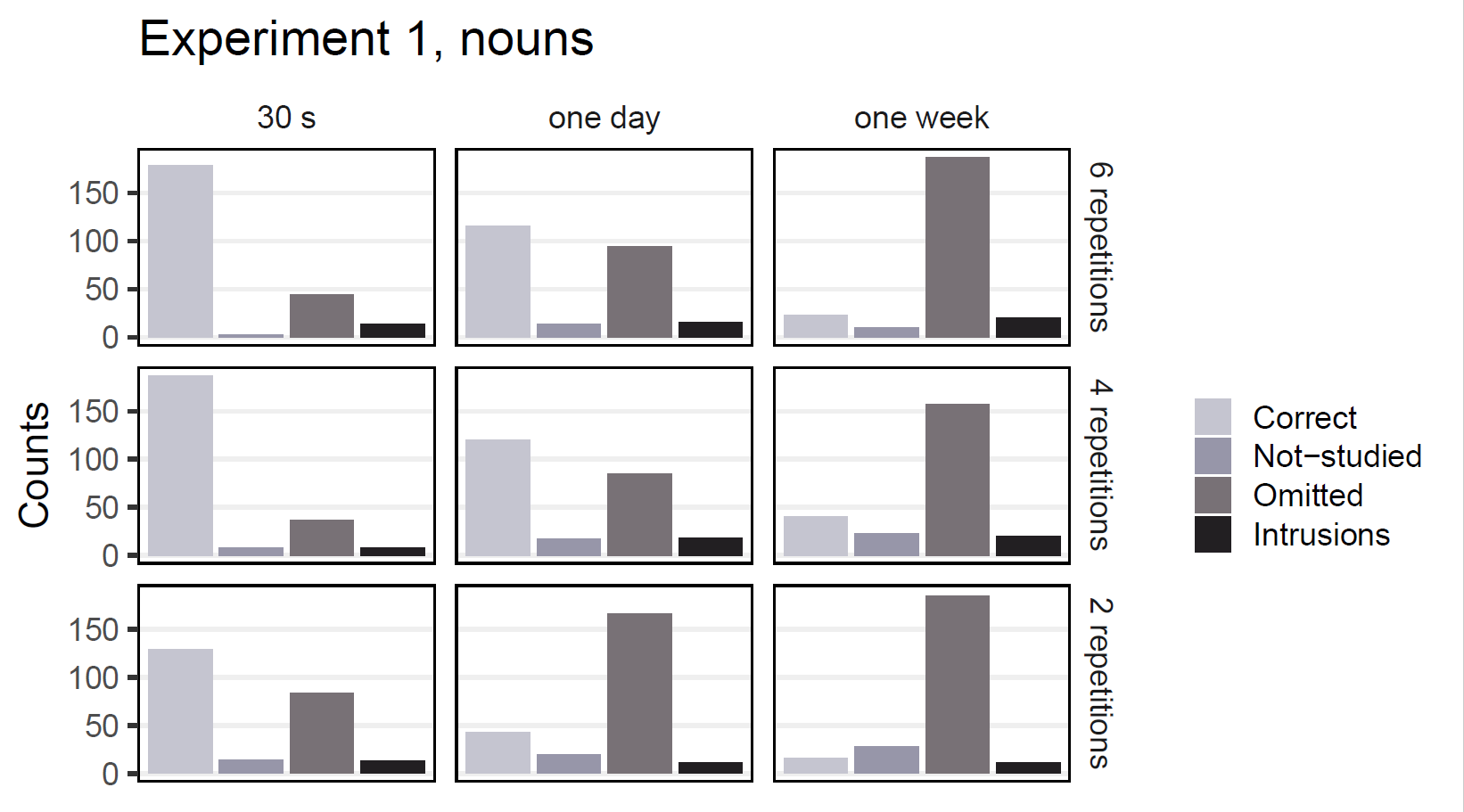


**Figure S3**

*Total of correctly and incorrectly recalled verbs in Experiment 2.*


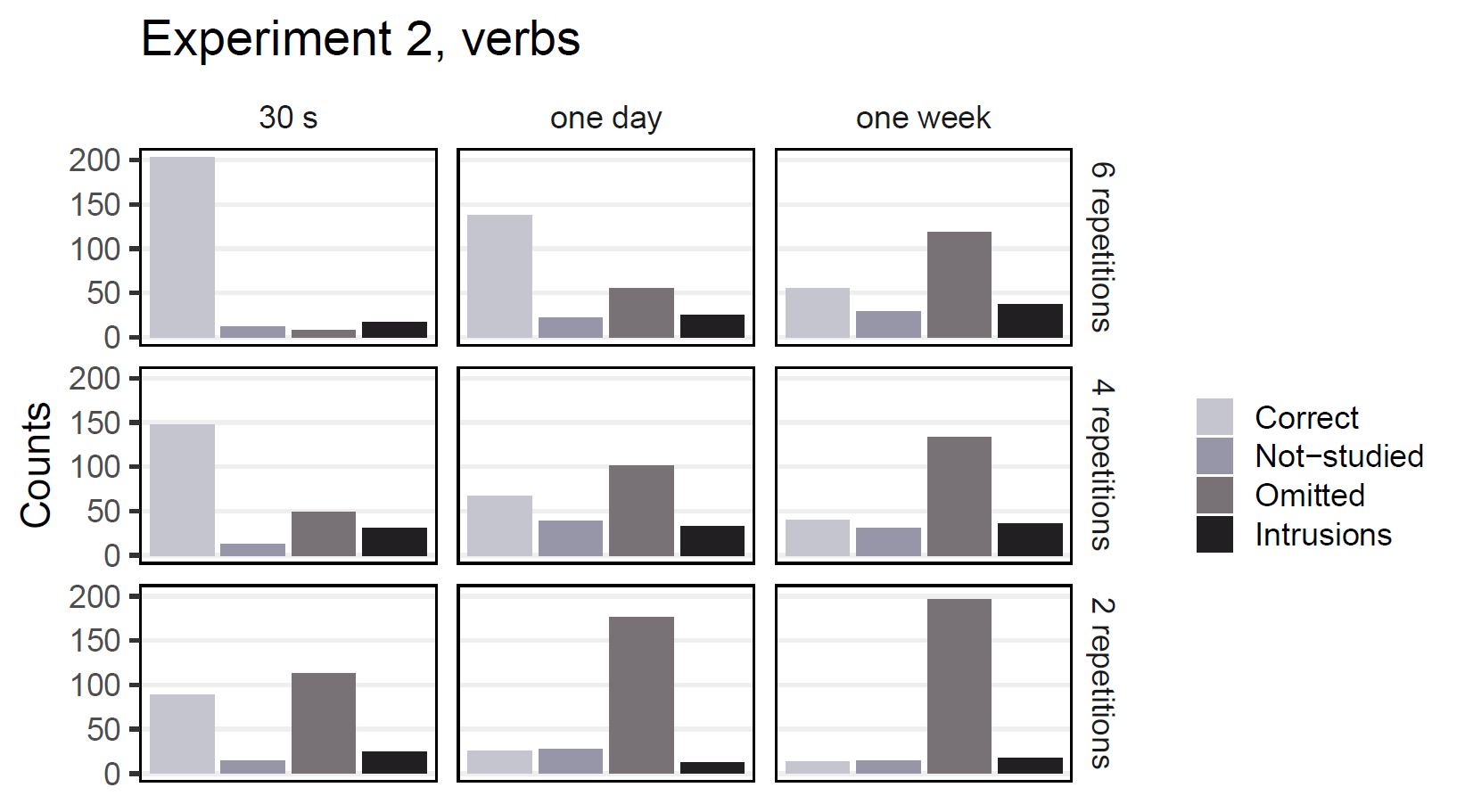


**Figure S4**

*Total of correctly and incorrectly recalled nouns in Experiment 2.*


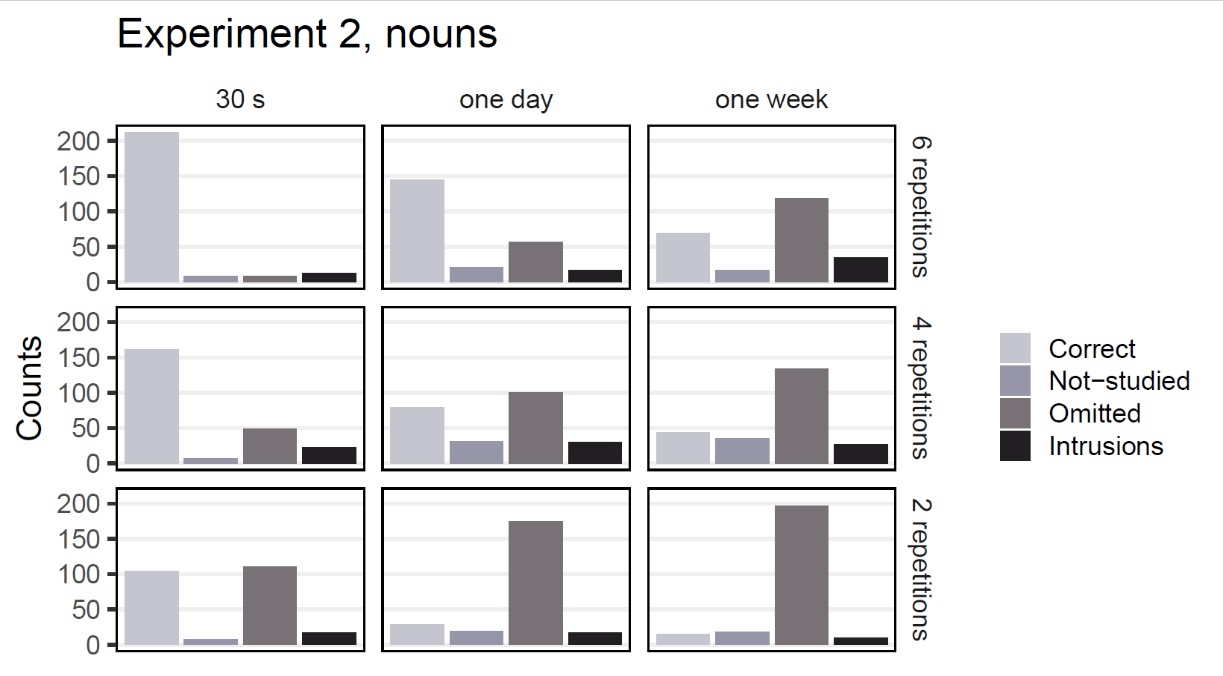


**Figure S5**

*Total of correctly and incorrectly recalled verbs in Experiment 3.*


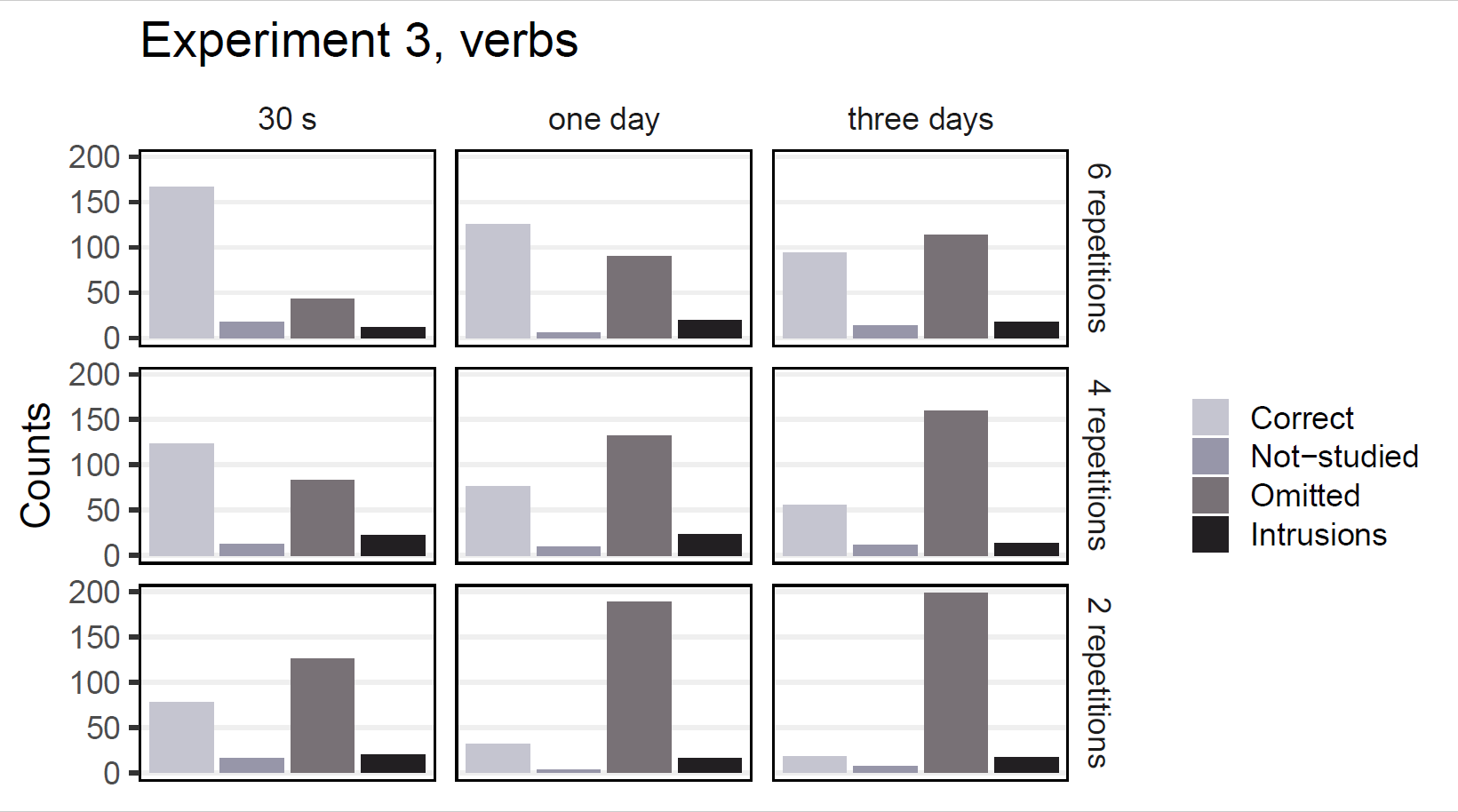


**Figure S6**

*Total of correctly and incorrectly recalled nouns in Experiment 3.*


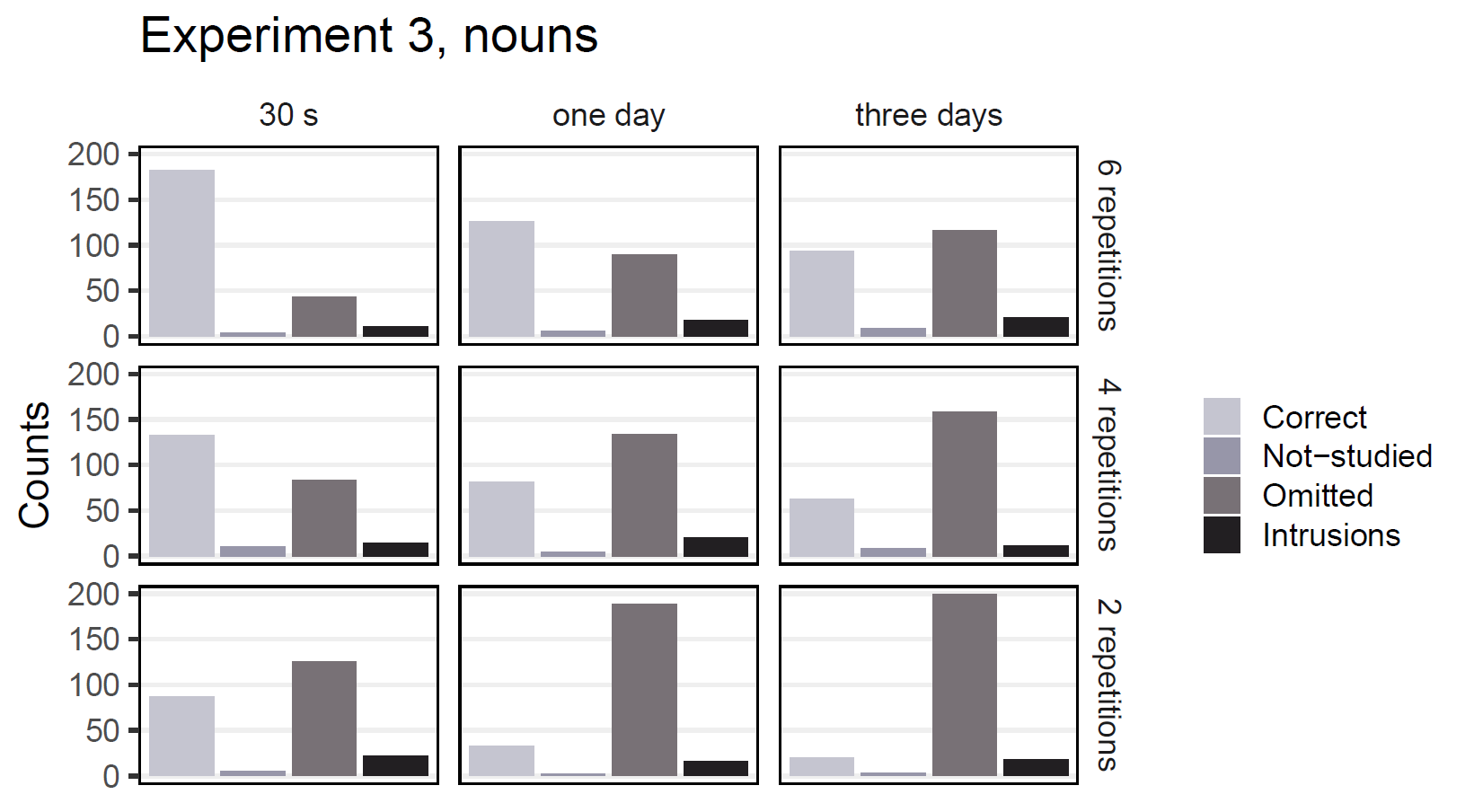


**Figure S7**

*Total of correctly and incorrectly recalled verbs in Experiment 4.*


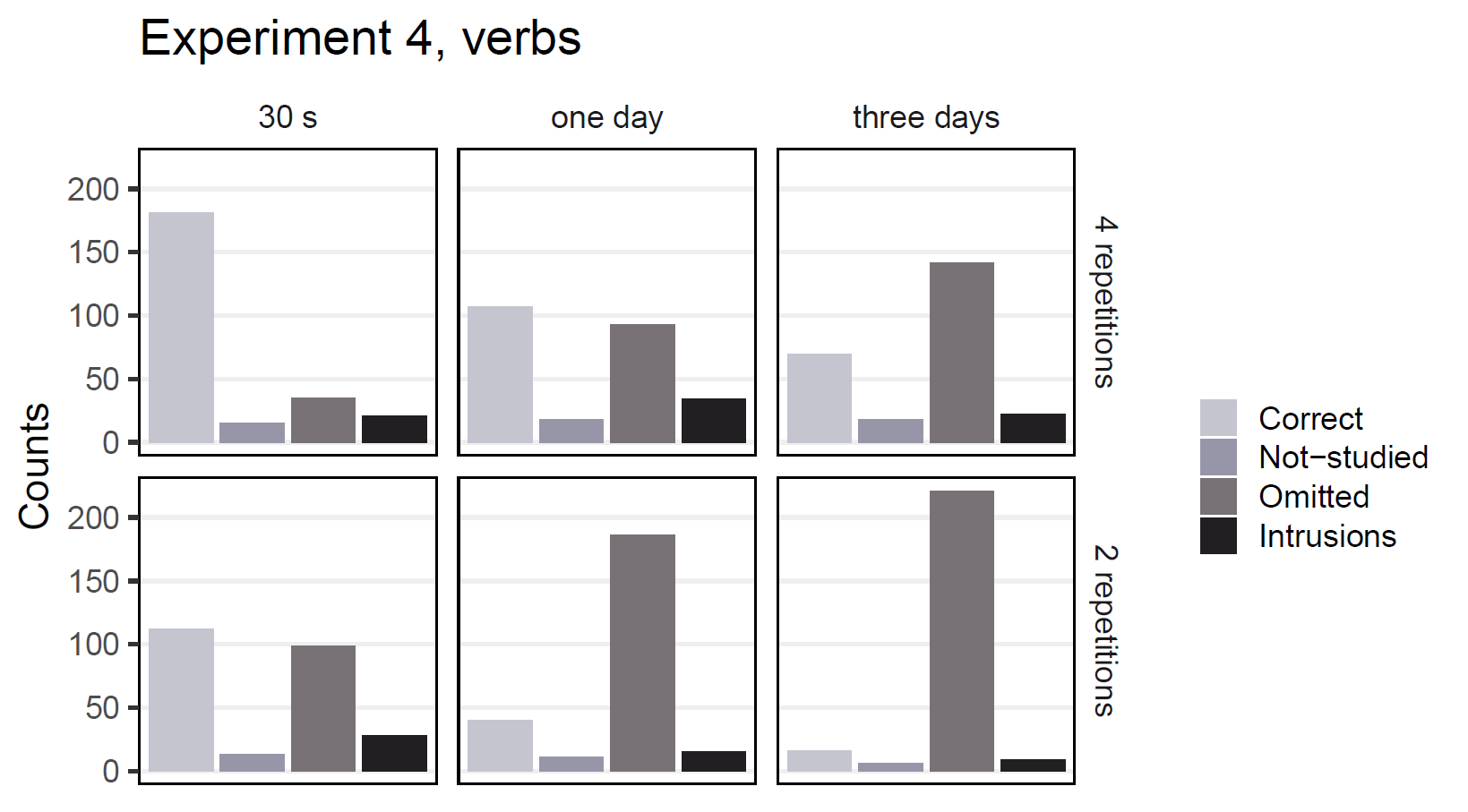


**Figure S8**

*Total of correctly and incorrectly recalled nouns in Experiment 4.*


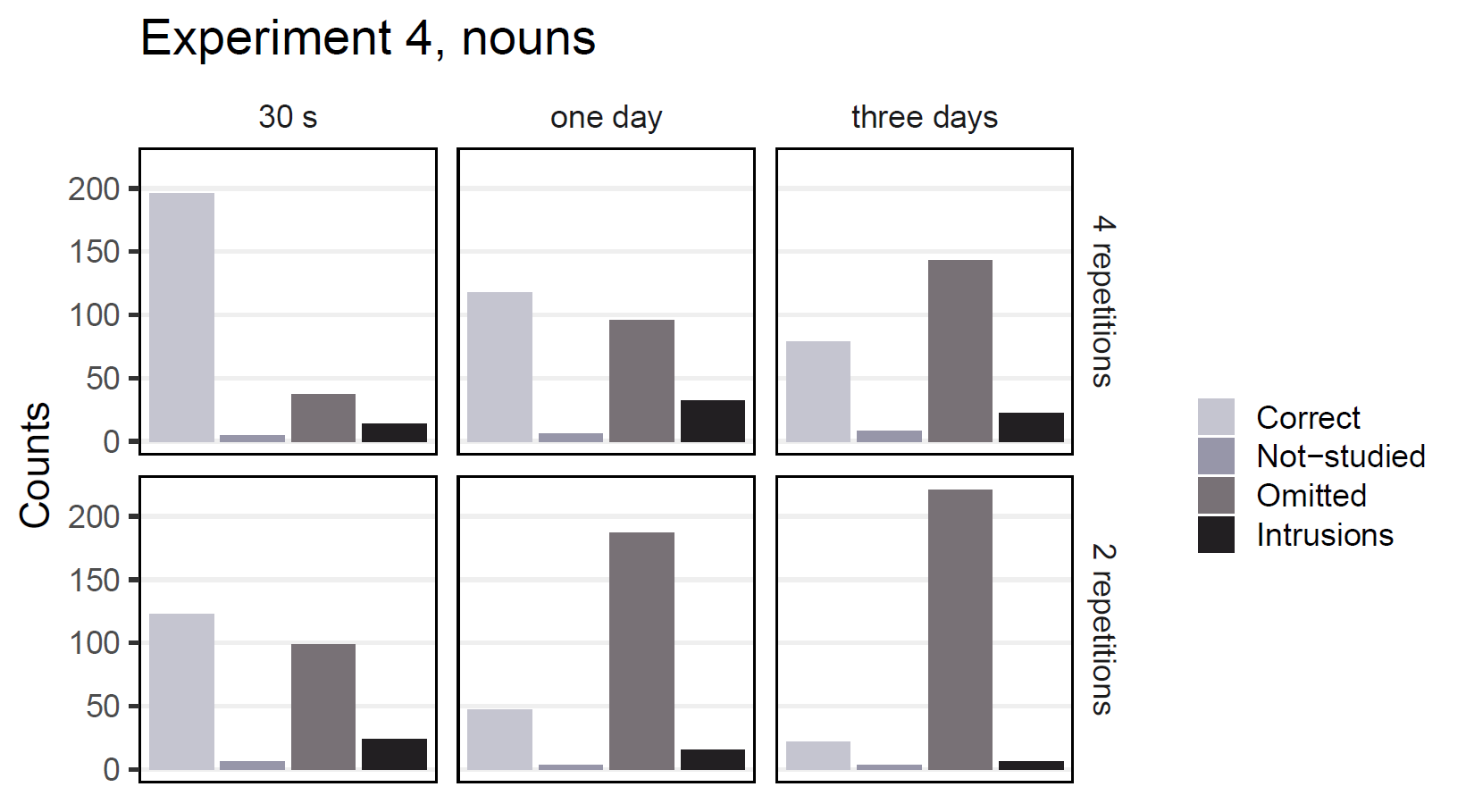


**Appendix C**

# Results with stringent scoring criterion

# For the following results, a response was considered correct when both the verb and the object were correct.

# Experiment 1

## **Retention interval effect**

There was substantial evidence of the retention interval effect between 30 s and 24 hr (b = -1.73, SD = 0.32, CI = [-2.37, -1.11]), between 30 s and 1 week (b = -4.98, SD = 0.5, CI = [-6.05, -4.07]), and between 24 hr and 1 week (b = -3.25, SD = 0.52, CI = [-4.35, -2.28]).

## **Effect of the number of repetitions**

There was no substantial evidence of a difference between six and four repetitions (b = 0.24, SD = 0.58, CI = [-0.88, 1.36]). There was substantial evidence of a difference between six and two repetitions (b = -1.56, SD = 0.6, CI = [-2.73, -0.42]), and between four and two repetitions (b = -1.80, SD = 0.61, CI = [-3.02, -0.61]).

## **Interactions**

There was no evidence of a difference in the forgetting slopes from 30 s to 24 hr between six and four repetitions (b = -0.36, SD = 0.41, CI = [-1.18, 0.45]), four and two repetitions (b = -0.53, SD = 0.49, CI = [-1.49, 0.41]), or six and two repetitions (b = -0.89, SD = 0.48, CI = [-1.85, 0.03]). There was evidence of a difference from one day to one week between six and four repetitions (b = 1.29, SD = 0.59, CI = [0.17, 2.46]) and six and two repetitions (b = 2.15, SD = 0.66, CI = [0.84, 3.45]), but not between four and two (b = 0.86, SD = 0.62, CI = [-0.3, 2.08]). Finally, we found evidence of a difference from 30 s to one week between six and two repetitions (b = 1.26, SD = 0.62, CI = [0.06, 2.49]), but not between six and four repetitions (b = 0.92, SD = 0.57, CI = [-0.13, 2.05]) or between four and two (b = 0.33, SD = 0.56, CI = [-0.76, 1.42]).

# Experiment 2

## **Retention interval effect**

There was substantial evidence of the retention interval effect between 30 s and 24 hr (b = -1.69, SD = 0.29, CI = [-2.28, -1.13]), between 30 s and 1 week (b = -3.74, SD = 0.43, CI = [-4.63, -2.91]), and between 24 hr and 1 week (b = -2.05, SD = 0.39, CI = [-2.86, -1.3]).

## **Effect of the number of repetitions**

There was substantial evidence of a difference between six and four repetitions (b = -1, SD = 0.41, CI = [-2.2, -0.56]), between six and two repetitions (b = -3, SD = 0.42, CI = [-3.53, -1.87]), and between four and two repetitions (b = -1, SD= 0.41, CI = [-2.12, -0.5]).

## **Interactions**

There was no substantial evidence of a difference in the forgetting slopes from 30 s to 24 hr between six and four repetitions (b = -0.22, SD = 0.39, CI = [-0.99, 0.55]), between four and two repetitions (b = -0.09, SD = 0.42, CI = [-0.93, 0.77]), or between six and two repetitions (b = -0.31, SD = 0.44, CI = [-1.19, 0.54]); There was no evidence of a substantial difference from 24 hr - 1 week between ix and four repetitions (b = 0.76, SD = 0.58, CI = [-0.44, 1.87]), between four and two repetitions (b = -0.45, SD = 0.72, CI = [-1.92, 0.9]), or between six and two repetitions (b = 0.31, SD = 0.73, CI = [-1.18, 1.65]); and finally, no substantial evidence of a difference from 30 s to 1 week between six and four repetitions (b = 0.54, SD = 0.6, CI = [-0.73, 1.69]), between four and two repetitions (b = -0.53, SD = 0.7, CI = [-1.98, 0.82]) or between six and two repetitions (b = 0, SD = 0.73, CI = [-1.55, 1.35]).

# Experiment 3

**Retention interval effect**

There was substantial evidence of the retention interval effect between 30 s and 24 hr (b = -0.9, SD = 0.25, CI = [-1.39, -0.41]), between 30 s and 1 week (b = -1.6, SD = 0.32, CI = [-2.26, -0.98]), and between 24 hr and 1 week (b = -0.7, SD = 0.31, CI = [-1.34, -0.09]).

**Effect of the number of repetitions**

There was no substantial evidence of a difference between six and four repetitions (b = -1, SD = 0.35, CI = [-1.45, -0.08]). There was substantial evidence of a difference between six and two repetitions (b = -1, SD = 0.34, CI = [-2.18, -0.84]), and between four and two repetitions (b = -1, SD= 0.36, CI = [-1.45, -0.04]).

**Interactions**

There was no substantial evidence of a difference from 30 s to 24 hours between six and four repetitions (*b* = -0.17, *SD* = 0.35, CI = [-0.87, 0.51]), four and two repetitions (*b* = -0.34, *SD* = 0.38, CI = [-1.09, 0.4]), or six and two repetitions (*b* = -0.51, *SD* = 0.38, CI = [-1.29, 0.23]). The same pattern was found from 24 hours to one week between six and four repetitions, (*b* = 0.16, *SD* = 0.43, CI = [-0.69, 1.01]), four and two repetitions (*b* = -0.51, *SD* = 0.51, CI = [-1.53, 0.48]) and six and two repetitions (*b* = -0.34, *SD* = 0.5, CI = [-1.34, 0.65]). Finally, no substantial evidence was found for a difference from 30 s to one week between six and four repetitions (*b* = -0.01, *SD* = 0.42, CI = [-0.84, 0.8]), four and two repetitions (*b* = -0.84, *SD* = 0.49, CI = [-1.84, 0.1]) or six and two repetitions (*b* = -0.85, *SD* = 0.48, CI = [-1.83, 0.03]).

# Experiment 4

# Effect of time

There was substantial evidence of the retention interval effect between 30 s and one day (b = -1.78, SD = 0.29, CI = [-2.36, -1.22]). 30 s and three days (b = -2.96, SD = 0.4, CI = [-3.8, -2.24]), and between one day and three days (b = -1.18, SD = 0.37, CI = [-1.95, -0.51])

# Effect of number of repetitions

There was substantial evidence of the difference between four and six repetitions (b = -2, SD = 0.5, CI = [-2.61, -0.62]).

# Interactions

There was no substantial evidence of a difference between four and two repetitions from 30 s to one day (b = -0.38, SD = 0.42, CI = [-1.24, 0.43]), from 30 s to three days (b = -0.75, SD = 0.55, CI = [-1.88, 0.24]), or from one day to three days (b = -0.37, SD = 0.55, CI = [-1.48, 0.68]).
